# Associations between 25-Hydroxyvitamin D and Immunologic, Metabolic, Inflammatory Markers in Treatment-Naive HIV-Infected Persons: The ANRS CO9 «COPANA» Cohort Study

**DOI:** 10.1371/journal.pone.0074868

**Published:** 2013-09-18

**Authors:** Camille Legeai, Corinne Vigouroux, Jean-Claude Souberbielle, Olivier Bouchaud, Faroudy Boufassa, Jean-Philippe Bastard, Robert Carlier, Jacqueline Capeau, Cécile Goujard, Laurence Meyer, Jean-Paul Viard, the ANRS-COPANA Cohort Study Group

**Affiliations:** 1 Institut National de la Santé et de la Recherche Médicale (INSERM), unité mixte de recherche et de service (UMRS) 1018, équipe « Epidémiologie du VIH et des infections sexuellement transmissibles », centre de recherche en épidémiologie et santé des populations (CESP)-INSERM U1018, Le Kremlin-Bicêtre, France; 2 Assistance Publique-Hôpitaux de Paris, hôpital Tenon, service de biochimie et hormonologie, Paris, France; 3 INSERM UMRS938, centre de recherche Saint-Antoine, Paris, France; 4 Université Pierre et Marie Curie-Paris 06, institute of cardiometabolism and nutrition (ICAN), Paris, France; 5 Assistance Publique-Hôpitaux de Paris, service d’explorations fonctionnelles, hôpital Necker, Paris, France; 6 Assistance Publique-Hôpitaux de Paris, service des maladies infectieuses, hôpital Avicenne, Bobigny, France; 7 Université Paris-Nord, Bobigny, France; 8 Assistance Publique-Hôpitaux de Paris, service de radiologie et imagerie médicale, hôpital Raymond-Poincaré, Garches, France; 9 Assistance Publique-Hôpitaux de Paris, service de médecine interne, hôpital Bicêtre, Le Kremlin-Bicêtre, France; 10 Université Paris-Sud, Le Kremlin-Bicêtre, France; 11 Assistance Publique-Hôpitaux de Paris, service d’épidémiologie et de santé publique, hôpital Bicêtre, Le Kremlin-Bicêtre, France; 12 Assistance Publique-Hôpitaux de Paris, centre de diagnostic et de thérapeutique, Hôpital Hôtel-Dieu, Paris, France; 13 Equipe d’accueil 3620, Université Paris Descartes, Paris, France; Alberta Provincial Laboratory for Public Health/University of Alberta, Canada

## Abstract

**Objectives:**

Low 25(OH)D has been associated with dyslipidemia, insulin resistance and inflammation in both general and HIV-infected (mostly treated) populations. We investigated these associations in antiretroviral-naïve HIV-infected persons.

**Design:**

We measured plasma 25(OH)D, metabolic, immunologic and inflammatory markers in 355 persons (204 Whites, 151 Blacks) at enrollment in the ANRS COPANA cohort.

**Methods:**

25(OH)D levels were categorized <10 ng/mL (severe deficiency) and <20 ng/mL (deficiency). Statistical analyses were adjusted for sampling season, ethnicity and the interaction between season and ethnicity.

**Results:**

25(OH)D insufficiency (<30 ng/mL), deficiency (<20 ng/mL) and severe deficiency (<10 ng/mL) were highly prevalent (93%, 67% and 24% of patients, respectively). Blacks had significantly lower 25(OH)D than Whites (median: 13 *vs.* 17 ng/mL, *P*<0.001), with markedly less pronounced seasonal variation. Smoking and drinking alcohol were associated with having a 25 OHD level<10 ng/mL. In patients with 25(OH)D<10 ng/mL, the proportion of persons with a CD4 count<100/mm^3^ was higher than in patients with 25(OH)D≥10 ng/mL (18.8% *vs.* 10.7%, *P* = 0.04). Persons with 25 OHD<10 ng/mL had higher levels of hsCRP (1.60 mg/L [IQR: 0.59–5.76] *vs.* 1.27 mg/L [0.58–3,39], *P* = 0.03) and resistin (16.81 ng/L [IQR: 13.82–25.74] *vs.* 11.56 ng/L [IQR: 8.87–20.46], *P* = 0.02), and, among Blacks only, sTNFR2 (2.92 ng/mL [2.31–4.13] *vs.* 2.67 ng/mL, [1.90–3.23], *P* = 0.04). The strength and significance of the association between CD4<100/mm^3^ and 25 OHD<10 ng/mL were reduced after adjustment on sTNFR1, sTNFR2, and hsCRP levels. In multivariate analysis, a CD4 count <100/mm^3^, resistin concentration and smoking were independently associated with 25(OH)D<10 ng/mL.

**Conclusions:**

Severe vitamin D deficiency was associated with low CD4 counts and increased markers of inflammation in ARV-naïve HIV-infected persons.

## Introduction

In the general population, vitamin D deficiency, assessed by low levels of 25-hydroxy vitamin D (25(OH)D), has been associated with diverse conditions, of which many have become of concern in the HIV-infected population, such as infections, cardiovascular disease, insulin resistance and diabetes, dyslipidemia, cancer, neurocognitive impairment, frailty, renal function alteration, osteopenia/osteoporosis, and autoimmune diseases [1]–[4]. It has also been associated with all-cause and cardiovascular mortality in persons weakened by age [5] or chronic conditions such as high cardiovascular risk [6], diabetes [7], cancer [8], heart transplantation [9] and renal failure [10].

Studies in HIV-infected persons, mostly antiretroviral-treated, showed that vitamin D deficiency is not more prevalent than in the general population [11], [12]. However, besides factors usually associated with vitamin D deficiency (e.g. phototype, season, region), HIV disease parameters such as a low CD4 count and exposure to antiretrovirals have been associated with low 25(OH)D levels [11]. Whatever the causes of lowered 25(OH)D in HIV-infected persons, the known associations of vitamin D deficiency with inflammation [13], [14], activation of coagulation [15], and impaired T-cell function [16] makes it a plausible cofactor in the pathogenesis of clinical complications and comorbidities of HIV infection. Indeed, vitamin D deficiency has been associated with clinical and pre-clinical endpoints in HIV-infected persons: all-cause mortality during untreated [17] and treated [18], [19] HIV infection, lesser CD4 cell gain on antiretroviral therapy [20], [21], AIDS and non-AIDS-defining events [18], [22], [23], insulin resistance [24], type 2 diabetes [25], and atherosclerosis [26]–[28]. Altogether, these data suggest that treated and untreated HIV-infected persons might be particularly susceptible to the deleterious effects of vitamin D deficiency.

The interpretation of studies in treated patients is hampered by the effects of antiretroviral drugs, their metabolic adverse events, and their possible impact on vitamin D metabolism [11]. The present study investigated for the first time the associations between 25(OH)D levels and body composition, metabolic, immunologic and inflammatory markers in a large group of White and Black antiretroviral-naïve HIV-1-infected persons enrolled soon after HIV diagnosis.

## Methods

### Study Design

The ANRS-C09-COPANA cohort is an ongoing prospective study conducted in 37 hospitals in France. Between 2004 and 2008, it enrolled 800 recently diagnosed (<1 year) HIV-1-infected adults, antiretroviral naïve at baseline, with semi-annual follow-up. The Paris-Cochin Ethics Committee approved the study protocol and all participants gave written informed consent. At enrollment and each scheduled visit, detailed sociodemographic, clinical and biological data were collected. Patients who accepted to participate entered a metabolic substudy comprising an oral glucose tolerance test (OGTT), measurements of inflammatory markers, insulin and adipokines, computed tomography (CT) at the level of the L4 vertebra and dual-energy X-ray absorptiometry (DEXA) [29]. 25(OH)D was measured in plasma samples taken at enrollment from 355 patients of Black or White ethnicity (patients in the metabolic study and patients selected for a longitudinal study on the basis of remaining treatment-naïve at 1 year of follow-up).

### Measurements

Age, gender, ethnicity, smoking, alcohol consumption, HIV transmission category, blood pressure, height, weight, body mass index (BMI) and waist circumference were documented at enrollment. CD4 cell count, HIV RNA viral load, blood levels of fasting lipids, glucose and creatinine, were measured at each center with standard procedures. The MDRD equation was used for calculation of the estimated glomerular filtration rate (eGFR). 25(OH)D was measured on cryopreserved plasma samples at Necker Hospital, Paris, using the DiaSorin® radioimmunassay. Sampling season was defined as summer/autumn for patients sampled in June – November, and as winter/spring for those sampled in December – May.In patients enrolled in the metabolic substudy, glycemia and insulinemia were measured at T0 and T120 minutes of an OGTT (75 g of glucose) performed after at least 8-hour fast. Inflammatory markers, insulin and adipokines levels were measured on cryopreserved serum samples at Tenon Hospital, Paris. Insulin was measured with a method avoiding cross-reactivity with pro-insulin (ARCHITECT system; Abbott, North Chicago, IL USA). Homeostasis model assessment of insulin resistance (HOMA-IR) values was calculated as [T0 insulin (mU/L) × T0 glucose (mmol/L)/22.5]. High sensitivity C-reactive protein (hsCRP) was determined by nephelometry on an IMMAGE analyser (Beckman-Coulter, Villepinte, France). Serum total adiponectin, resistin, leptin, interleukin (IL)-6, monocyte chemoattractant protein-1 (MCP-1), tumor necrosis factor (TNF)-α and its soluble receptors sTNFR 1 and 2 were measured with multiplexed bead-based immunoassays (Linco Research Inc., St Charles, MO, USA and Biosource International Inc.; Camarillo, CA, USA), with respective detection limits of 145.6, 6.7, 85.4, 1.6, 0.14, 0.14, 15 and 15 pg/mL, on a Bio-Plex 200 system (Bio-Rad laboratories Inc., Hercules, CA, USA) using BioPlex Manager TM 3.0 software.

Subcutaneous and visceral adipose tissue areas (SAT and VAT) were calculated in the same radiological center, from 1-cm reconstructed slices of abdominal L4 CT scans, using an Extended Brilliance workstation and QCTA software (EBW, QCTA, Philips Medical Systems, Eindhoven, the Netherlands). DEXA was performed using Lunar Prodigy (GE Medical Systems, Madison, WI, USA) or Hologic (Hologic, Inc., Bedford, MA, USA) densitometers and percentages of total, trunk and limb fat were recorded.

### Statistical Analysis

Continuous variables were reported as medians and 25^th^ to 75^th^ percentiles (IQR), and compared according to baseline characteristics by using non-parametric Wilcoxon tests. Categorical variables were reported as percentages and compared by using χ^2^ or Fisher’s tests. The LOESS method was used to plot a smooth curve showing 25(OH)D level variations according to sampling season in White and Black patients. Multiple linear regression models were used to explore association between 25(OH)D levels and ethnicity and sampling season (*P*-values were estimated by the Wald tests).

The analysis performed within the EuroSIDA study showed that increased risks of AIDS-defining events and all-cause death were associated with very low 25(OH)D levels, <12 ng/mL, which is very close to the commonly accepted definition of severe deficiency (<10 ng/mL) [18]. We therefore decided to classify patients into 2 categories according to severely deficient status (25(OH)D<10 ng/mL and ≥10 ng/mL). We also classified patients according to deficient status (25(OH)D<20 ng/mL and ≥20 ng/mL).

Associations between 25(OH)D severely deficient or deficient status and socio-demographic characteristics, immuno-virologic, inflammatory and metabolic markers were explored using logistic regression models; *P*-values were estimated by the Wald tests, and analyses were systematically adjusted for season, ethnicity and season-ethnicity interaction. Multivariate analysis was performed by logistic regression. All variables associated with severe vitamin D deficiency with a *P* value≤0.10 after adjustment for season, ethnicity and season-ethnicity interaction were included in the initial model, except visceral adipose tissue measurement due to the statistical power issue. Backward stepwise selection process of variables with *P*<0.10 was used to determine the final multivariate logistic model; season, ethnicity and season-ethnicity interaction were forced into the model.

We further explored whether the association between severe immunodeficiency and low levels of 25(OH)D could be explained by increased levels of inflammatory markers. To do so, we used a multivariate logistic model assessing the association between severe immune deficiency (as the dependent variable) with severe vitamin D deficiency and inflammatory markers. Several models were built, one per inflammatory marker.

Statistical analyses were performed using SAS software version 9.2 (SAS Institute, Cary, NC, USA).

## Results

### Baseline Characteristics

Among the 355 patients, 108 (30%) were women and 150 (42%) were men having sex with men (MSM), 97% of patients having been infected by the sexual route. At enrollment, median age was 36 years (IQR: 30–44) and median BMI was 23.0 kg/m^2^ (IQR: 20.8–25.7). Median duration since HIV diagnosis was 3.8 months (IQR: 1.5–7.1), median HIV-1 RNA level was 4.6 log_10_ copies/mL (IQR: 4.1–5.2), median CD4+ T-cell count was 300/mm^3^ (IQR: 173–463) and 34 patients (9.6%) were at CDC stage C of infection. 151 (43%) patients were Black (134 of African and 17 of Afro-Caribbean origin). Compared to Whites, Black patients were younger (median age: 34 *vs.* 37 years, *P* = 0.03), more often female (55% *vs.* 12%, *P*<0.001) or heterosexual men (70% *vs.* 24% of the men, *P*<0.001), they had a higher BMI (median: 23.9 *vs.* 22.5, *P*<0.001) and their median CD4+ T-cell count was lower (245 *vs.* 344/mm^3^, *P*<0.001).

### Low 25(OH)D Concentrations in Antiretroviral-naïve, Recently Diagnosed HIV-infected Patients

A vast majority of the patients (93%) had vitamin D insufficiency, *i.e.* 25(OH)D<30 ng/mL. Criteria for vitamin D deficiency (<20 ng/mL) or severe deficiency (<10 ng/mL) were met by 67 and 24% of the patients, respectively.

Patients sampled in winter/spring had a much lower 25(OH)D level than those sampled in summer/autumn: median of 13 ng/mL (IQR: 9–19.5) *vs.* 18 ng/mL (IQR: 12–24) (*P*<0.001). Median 25(OH)D was significantly lower in Black than in White patients, even after adjustment for age, sex, sampling season and BMI: adjusted mean 13.9 ng/mL (95%CI: 12.5–15.3) in Blacks *vs.* 18.7 ng/mL (95%CI:17.3–20.1) in Whites, (*P*<0.001).

**Figure 1:**
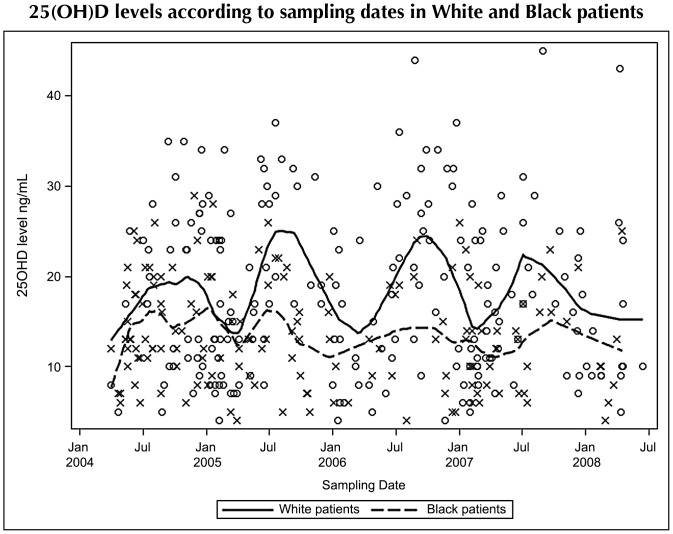
25(OH)D plasma levels according to sampling dates in White and Black patients. Jan: January; Jul: July O and X symbols depict individual 25(OH)D plasma levels in White and Black patients, respectively.

As illustrated by figure 1, season’s impact on 25(OH)D plasma concentrations was markedly less pronounced in Black than in White patients. In a linear regression model, season (*P*<0.001), ethnicity (*P*<0.001) and the interaction between season and ethnicity (*P*<0.01) were significantly associated with 25(OH)D levels. Indeed, the difference in means between the seasons was 6.4 in Whites *vs.* 2.2 ng/mL in Blacks. This difference remained significant after taking into account the residence zone (South *vs.* North of France).

### Association between Severe Vitamin D Deficiency, Clinical Characteristics and Biomarkers (Table 1)

**Table 1:** Characteristics of patients according to severe vitamin D deficiency.

Variables expressed as median [IQR] or N (%)		N		25-hydroxyvitamin D categories				P value *	
				<10 ng/mL		≥10 ng/mL			
Sampling during Winter/Spring		200		60 (70.6)		140 (51.9)			
Sampling during Summer/Autumn		155		25 (29.4)		130 (48.1)		<0.01	
Age (years)	355		35 [30]–[44]		36 [30]–[43]		0.92		
Male	247		59 (69.4)		188 (69.6)				
Female	108		26 (30.6)		82 (30.4)		0.76		
Non MSM		210		58 (68.2)		152 (56.3)			
MSM		145		27 (31.8)		118 (43.7)		0.08	
Non smokers	236		50 (58.8)		186 (69.1)				
Smokers	118		35 (41.2)		83 (30.9)		0.02		
Alcohol intake <20 g/day	293		68 (85.0)		225 (90.4)				
Alcohol intake ≥20 g/day	36		12 (15.0)		24 (9.6)		0.05		
Waist circumference (cm)		325		82 [75.5–87.5]		83 [77.0–90.0]		0.40	
Body mass index (BMI)		347		22.1 [20.3–24.7]		23.1 [21.1–25.9]		0.11	
BMI <30		325		79 (96.3)		246 (92.8)			
BMI ≥30		22		3 (3.7)		19 (7.2)		0.26	
Total body fat assessed by DEXA (%)		106		19.58 [11.9–30.0]		20.8 [14.0–28.9]		0.36	
SAT assessed by CT scan (cm^2^)		147		126.1 [73.8–192.8]		140.4 [82.5–196.5]		0.36	
VAT assessed by CT scan (cm^2^)		147		66.8 [43.3–75.9]		71.3 [44.0–112.9]		0.02	
eGFR, MDRD formula (mL/min/1.73 m^2^)		343		102.9 [87.5–120.2]		95.7 [84.3–110.7]		0.26	
Plasma HIV RNA (log_10_ copies/mL)		349		4.7 [4.0–5.21]		4.6 [4.0–5.1]		0.87	
Baseline CD4 count (cells/mm^3^)		355		288 [162–488]		300 [179–437]		0.72	
Patients with CD4 count <100/mm^3^		45		16 (18.8)		29 (10.7)			
Patients with CD4 count ≥100/mm^3^		310		69 (81.2)		241 (89.3)		0.04	
Patients with CD4 count <200/mm^3^		101		29 (34.1)		72 (26.7)			
Patients with CD4 count ≥200/mm^3^		254		56 (65.9)		198 (73.3)		0.13	
IL-6 (pg/mL)		196		2.87 [0.92–7.15]		2.74 [1.04–5.06]		0.80	
hsCRP (mg/L)		201		1.60 [0.59–5.76]		1.27 [0.58–3.39]		0.03	
TNF-α (pg/mL)		198		5.63 [3.72–7.89]		6.78 [4.30–9.41]		0.07	
sTNFR-1 (ng/mL)		196		1.84 [1.50–2.27]		1.72 [1.28–2.16]		0.10	
sTNFR-2 (ng/mL)		196		2.59 [1.96–3.21]		2.56 [1.98–3.23]		0.47	
MCP-1 pg/mL)		200		206.7 [143.5–317.7]		218.3 [153.9–334.8]		0.78	
Resistin (ng/L)		125		16.81 [13.82–25.74]		11.56 [8.87–20.46]		0.02	
Leptin (µg/L)		198		3.52 [0.77–6.84]		3.96 [1.48–10.87]		0.10	
Adiponectin (mg/L)		171		14.56 [10.54–18.56]		14.71 [9.07–18.25]		0.17	
Triglycerides (mmol.L)		327		0.96 [0.63–1.65]		0.97 [0.67–1.40]		0.30	
Total cholesterol (mmol/L)		330		4.30 [3.74–4.78]		4.27 [3.64–4.90]		0.81	
HDL cholesterol (mmol/L)		321		1.11 [0.92–1.31]		1.11 [0.87–1.37		0.79	
LDL cholesterol (mmol/L)		320		2.61 [2.10–2.98]		2.60 [2.09–3.21]		0.50	
Glycemia (mmol/L), fasting		324		4.6 [4.3–5.0]		4.8 [4.4–5.2]		0.15	
Glycemia (mmol/L), OGTT t120		179		5.2 [4.6–5.7]		5.0 [4.2–5.8]		0.93	
Insulinemia (mU/L), fasting		202		5.6 [3.2–8.6]		5.3 [3.8–8.2]		0.96	
Insulinemia (mU/L), OGTT t120		166		19.5 [8.4–32.3]		17.85 [9.3–31.3]		0.84	
HOMA-IR index		190		1.19 [0.70–1.98]		1.09 [0.80–1.72]		0.75	

* except for the season variable, *P* values obtained from models including season, ethnicity, season-ethnicity interaction, and the considered variable.

IQR: interquartile range, NA: not applicable, DEXA: dual energy X-ray absorptiometry, SAT: subcutaneaous adipose tissue, VAT: visceral adipose tissue, CT: computed tomography, eGFR: estimated glomerular filtration rate, IL-6: interleukin 6, hsCRP: high sensitivity C-reactive protein, TNF: tumor necrosis factor, sTNFR: soluble TNF receptor, OGTT t120: oral glucose tolerance test (sample taken 120 minutes after glucose administration), HOMA-IR: homeostatic model assessment of insulin resistance.

Tobacco smokers and persons drinking more than 20 g of alcohol a day were significantly overrepresented among patients with severe vitamin D deficiency. The same trend, not reaching statistical significance (*P* = 0.08), was noted for non-MSM, compared with MSM.

Patients with 25(OH)D concentrations below 10 ng/mL had a more severe immune deficiency than patients with 25(OH)D≥10 ng/mL, since 18.8% *vs.* 10.7% had a CD4 count<100/mm^3^ (*P* = 0.04). They also had higher levels of the inflammatory markers hsCRP (1.60 mg/L [IQR: 0.59–5.76] *vs.* 1.27 mg/L [0.58–3,39], *P* = 0.03), and resistin (16.81 ng/L [13.82–25.74] *vs.* 11.56 ng/L [8.87–20.46], *P* = 0.02), and they tended to have lower TNF-**α** levels, but this did not reach significance (*P* = 0.07).

Patients with 25(OH)D<10 ng/mL also had a lower amount of VAT: 66.8 cm^2^ [43.3–75.9] *vs.* 71.3 cm^2^ [44.0–112.9] (*P* = 0.02), although BMI, total body fat, serum leptin and adiponectin were not significantly different as compared to the other patients.

Persons with plasma 25(OH)D≥10 ng/mL had a lower estimated glomerular filtration rate (eGFR) (95.7 mL/min/1.73 m^2^ [84.3–110.7] *vs.* 102.9 mL/min/1.73 m^2^ [87.5–120.2], unadjusted *P* = 0.02, Wilcoxon’s rank test). However, we did not find a dose-effect curve for this marker. Indeed, persons with plasma 25(OH)D≥20 ng/mL were those who more often had an eGFR lower than 90 mL/min/1.73 m^2^ (OR [95%CI]: 1.70 [1.03–2.80], *P* = 0.04, adjusted for season and ethnicity).

In multivariate analysis, a CD4 count below 100/mm^3^, resistin concentration and being a smoker remained independently associated with severe vitamin D deficiency.

There was no significant association between 25(OH)D severe deficiency and other markers, including glycemia, insulinemia, triglycerides, and total, HDL or LDL cholesterol.

Since 25(OH)D concentrations were significantly lower, with much less seasonal variations, in Black persons, we examined whether ethnicity modified the relations between 25(OH)D and the variables we examined. There was a significant interaction (*P* = 0.04) between ethnicity and sTNFR2 concentration, which was, in Black patients only, significantly higher in the severely vitamin D deficient group compared to patients with 25(OH)D≥10 ng/mL (in Blacks: 2.92 ng/mL [2.31–4.13] *vs.* 2.67 ng/mL, [1.90–3.23], *P* = 0.04; in Whites: 2.29 ng/mL [1.53–2.71] *vs.* 2.49 ng/mL [2.02–3.18], *P* = 0.38).

### Low CD4 Counts, Severe Vitamin D Deficiency, and Inflammatory Markers

We further investigated the relationship between severe immune deficiency (CD4<100/mm^3^) and severe vitamin D deficiency by testing different models comprising vitamin D (<10 ng/mL *vs.* ≥10 ng/mL) and several inflammatory markers. After adjustment for either hsCRP, sTNFR1 or sTNFR2, the significance and magnitude of the association between severe vitamin D deficiency and having a CD4 count<100/mm^3^ were reduced (Table 2), suggesting that these markers could in part explain this association. On the other hand, adjustment for resistin, IL-6, MCP-1 and TNF-**α** did not weaken the relationship between vitamin D deficiency and low CD4 counts.

**Table 2:** Association between severe immune deficiency (CD4<100/mm^3^) and severe vitamin D deficiency (<10 ng/mL) : odds ratio of severe immune deficiency after adjustment for different inflammatory markers (N = 201 patients with inflammatory markers measurements).

25(OH)D(ng/mL)	Covariate															
	None		hsCRP		sTNFR1		sTNFR2		MCP-1		TNF-α		IL-6		Resistin	
	OR (95%CI)	*P*	OR (95%CI)	*P*	OR (95%CI)	*P*	OR (95%CI)	*P*	OR (95%CI)	*P*	OR (95%CI)	*P*	OR (95%CI)	*P*	OR (95%CI)	*P*
≥10	1		1		1		1		1		1		1		1	
<10	2.95 (1.05–8.25)	0.04	2.40 (0.82–7.00)	0.11	2.21 (0.74–6.61)	0.16	2.46 (0.80–7.61)	0.12	2.96(1.04–8.40)	0.04	3.71 (1.26–10.90	0.02	2.89 (1.03–8.10)	0.04	3.06 (1.00–9.42)	0.05

Odds ratios, 95% CI and P values were estimated by multinomial logistic regressions adjusted for season, ethnicity and season.

#### Analysis of patients with vitamin D deficiency (25(OH)D<20 ng/mL)

When classifying patients as vitamin D-deficient (25(OH)D<20 ng/mL) *vs.* non-deficient (25(OH)D≥20 ng/mL), the analysis confirmed that higher 25(OH) levels were associated with lower eGFR (cf. supra). Although showing the same trends, none of the other relationships described above remained statistically significant (data not shown). On the other hand, leptin concentration was significatly higher in vitamin-D deficient patients (3.63 µg/L [1.20–9.58] *vs.* 3.39 µg/L [1.65–10.55], *P* = 0.03], which was not observed when using a 25(OH)D cut-off of 10 ng/mL).

### Longitudinal Study

Values of inflammatory and metabolic markers were available at one year of follow-up in a subgroup of 53 patients who remained antiretroviral-naïve. Patients with 25(OH)D <10 ng/mL at inclusion had higher levels of sTNFR1 and tended to have higher levels of hsCRP one year later than the other patients: 1.93 ng/mL [1.63–2.33] *vs.* 1.46 ng/mL [1.03–2.02] (*P* = 0.08) and 2.05 mg/L [1.19–4.86] *vs.* 1.24 mg/L [0.61–1.73] (*P* = 0.09), respectively.

## Discussion

We show herein that severe vitamin D deficiency is associated with very low CD4 counts (<100/mm^3^) and inflammation in untreated HIV infection. HIV infection is a risk factor for metabolic abnormalities and cardiovascular disease [30]. Antiretroviral drugs may play an additional role in the occurrence of these conditions, as shown by the risk for myocardial infarction conveyed by the exposure to protease inhibitors [31]. Research has recently focused on the role of HIV infection itself, treated or not, since inflammation and T-cell/monocyte activation have been associated with pre-clinical and clinical cardiovascular endpoints [30], [32]. Untreated HIV infection leads to an unfavorable lipid profile [33] and we have shown that immune deficiency in treatment-naïve patients is associated with insulin resistance [29]. Since vitamin D insufficiency has been associated with metabolic abnormalities, inflammation, and pre-clinical markers of atherosclerosis in both the general and HIV-infected populations, we explored the relations between 25(OH)D levels and body composition, metabolic, immunologic, and inflammatory markers in recently diagnosed antiretroviral-naïve HIV-infected patients.

### 25(OH)D Low Levels in Black and White Patients

Vitamin D insufficiency, deficiency, and severe deficiency, were frequent in this population of antiretroviral-naïve patients (found in 93%, 67% and 24% of cases, respectively). Low 25(OH) levels were associated with smoking in the multivariate analysis, as previously reported [34]. The 25(OH)D concentration was much lower in Black than in White persons. Interestingly, while the expected association between the level of 25(OH)D and the season of sampling was confirmed in Black as well as White patients, seasonal variation was much less pronounced in Black persons. Since it is not known whether the influence of a low 25(OH)D level on cell functions and blood markers is short- or mid/long-term, this could put Black persons at a higher risk of deleterious consequences of vitamin D deficiency, because smaller variations around the year reduce the probability of reaching desirable 25(OH)D levels.

### Association of Severe Vitamin D Deficiency Levels with Low CD4 Counts and Inflammation

We confirm an inverse relation between 25(OH)D levels and immunodeficiency [12], with the proportion of persons with a CD4 count below 100/mm^3^ being higher in persons with 25(OH)D<10 ng/mL. This finding, in a population where there is no influence of antiretrovirals on the CD4 level, could be explained by the role played by the vitamin D/vitamin D receptor system in activation and TCR signaling in naive T cells [16], and fits well with data showing that vitamin D deficiency is associated with clinical progression of untreated [17] or treated [18], [19] HIV disease, including AIDS-defining events [18], and with a poorer CD4 restoration on treatment [20], [21].

The strength and significance of the association between CD4<100/mm^3^ and 25 OHD<10 ng/mL were reduced after adjustment on sTNFR or hsCRP level. It is therefore possible to hypothesize that the association between severe vitamin D deficiency and immune deficiency may be mediated by uncontrolled inflammation, which should be tested in intervention trials.

Indeed, in patients with very low 25(OH)D levels, we found higher levels of the inflammatory marker hsCRP, and of resistin, which is also considered as an inflammatory marker in human, in addition to its promoting role on insulin resistance and cardiovascular diseases [35]. Very low 25 OHD levels at inclusion in the cohort were also associated with higher levels of hsCRP and sTNFR1 after one year of follow-up without treatment. Interestingly these associations were not found in patients with vitamin D deficiency (25(OH)D<20 ng/mL), which suggests a threshold effect rather than a dose-response curve.

Vitamin D deficiency has already been associated with higher levels of inflammatory markers in different conditions (patients at a high cardiovascular risk or with arthritis, older persons) [6], [13]. In placebo-controlled studies, vitamin D supplementation has been shown to lower the level of the inflammatory cytokine TNF-**α** and to increase the level of the anti-inflammatory cytokine IL-10, in patients with heart insufficiency [14], and to lower markers of inflammation and immune cell activation in tuberculosis [36]. Since cross-sectional studies such as the one presented here do not allow any conclusion on the temporality or causality of the relationships, the impact of vitamin D status on pro-inflammatory markers in HIV infection should be further studied, in intervention trials.

### 25(OH)D, Lipids and Insulin Resistance

We found no association between triglyceride levels, cholesterol or its fractions, glycemia, insulinemia, and severe vitamin D deficiency (<10 ng/mL) or deficiency (<20 ng/mL). Previous cross-sectional studies showed an inverse relation between vitamin D levels and lipids, but intervention studies have given divergent results regarding the effect of vitamin D supplementation on serum lipids [37].

A previously published study, involving mainly Caucasian patients treated with antiretrovirals, found a correlation between low 25(OH)D and high insulin levels, but not lipoatrophy or hypoadiponectinemia [24]. In contrast, in this antiretroviral-naïve study population, we found no significant relation between 25(OH)D and fasting insulinemia. Antiretroviral drugs and their metabolic side effects could explain this discrepancy.

### 25(OH)D, Body Composition and Adipokines

In patients with 25(OH)D<10 ng/mL, we observed lower values of VAT, while there was no association with BMI, leptin or adiponectin. On the other hand, patients with 25(OH)D levels in the deficient range (<20 ng/mL) had significantly higher blood leptin.

Vitamin D, being liposoluble, can be trapped in adipose tissue, explaining why obesity is associated with low 25(OH)D levels, which transiently increase after bariatric surgery, probably reflecting 25(OH)D release during weight loss [38]. On the other hand, severe malnutrition can be associated with low 25(OH)D. In the setting of HIV infection, a study, in mostly treated patients, showed a trend towards lower 25(OH)D levels in persons both in the lower and higher BMI tertiles [18]. Thus, the relationships between vitamin D levels, fat mass and adipokine production are probably complex and dynamic: 1–25(OH)_2_D has been shown to attenuate adiponectin production [39], and to suppress leptin secretion [40] in cultured human adipocytes, while leptin has been shown to stimulate fibroblast growth factor 23 expression and to suppress 1α-hydroxylation of vitamin D in a murine model [41]. This network of interactions may explain why no straightforward relationship was observed between 25(OH)D and adiposity or adipokine levels in this cross-sectional of HIV-infected persons, in whom ongoing inflammation also probably interferes with adipocyte functions.

### 25(OH)D and Renal Function

We found a trend towards an inverse association between 25(OH)D levels and MDRD-estimated GFR in treatment-naïve patients. This finding has been previously reported in HIV-infected patients, but a large majority was treated with antiretrovirals [11], [34]. In a setting where there is no influence of antiretrovirals on kidney function, this intriguing finding could be related to a recently described effect of vitamin D receptor activation, which was shown to increase the production of creatinine, without modification of its clearance [42].

### Strengths and Limitations of This Study

This is the first study assessing the association between 25(OH)D concentration and a panel of immunologic and metabolic markers, in a large group of recently diagnosed treatment-naïve persons living with HIV. In previous studies on vitamin D and HIV infection, a large majority of patients were receiving antiretroviral therapy, which could modify vitamin D metabolism, either directly, through the inhibition or activation of hydroxylation enzymes (protease inhibitors, efavirenz) [12], or undirectly, through the impact on proximal tubular function (tenofovir) [43]. Treatment of HIV infection also results in immune restoration, and, to a certain extent, in the control of inflammation, while antiretroviral treatment may interfere with lipid/glucose metabolism, kidney and adipocyte physiology.

A limitation of observational studies such as the present one is that normal or desirable values for 25(OH)D are a controversial issue, particularly when examining nonskeletal markers [4]. Severe vitamin D deficiency (25(OH)D<10 ng/mL) has been associated with a poorer clinical evolution of HIV infection in a previous study that showed a clear that different threshold 25(OH)D levels could be associated with different biological markers or clinical endpoints. Lastly, this cross-sectional analysis does not allow to conclude on the direction and causality of the observed relationships.

### Conclusion

In the absence of antiretroviral treatment, vitamin D deficiency was highly prevalent in White and Black HIV-infected patients living in France. In this population, low 25(OH)D was associated with smoking, with lower CD4 counts and higher inflammatory markers. We also found that the influence of season on vitamin D status was different in Black and White persons and that the level of the inflammatory marker sTNFR2 was associated with severe vitamin D deficiency only in Black persons. This could reflect differences in body composition, in the allelic distribution of genes related to vitamin D metabolism [44] or in the sensitivity to vitamin D [45]. This difference should be taken into account in future studies.

## Supporting Information

File S1 Members of the ANRS COPANA Cohort Study Group.
(DOC)
