# Supplementary material for: Associations between 25-Hydroxyvitamin D and Immunologic, Metabolic, Inflammatory Markers in Treatment-Naive HIV-Infected Persons: The ANRS CO9 «COPANA» Cohort Study
Source: PLoS One. 2013 Sep 18;8(9):e74868. doi: 10.1371/journal.pone.0074868 (PMC3776742; doi:10.1371/journal.pone.0074868)
Supplement: File S1 — Members of the ANRS COPANA Cohort Study Group. (DOC) [file pone.0074868.s001.doc]

Supporting information**: Members of the ANRS COPANA Cohort Study Group**

Christine Rouzioux and Véronique Avettand-Fenoël, Service de Virologie; Claudine Duvivier and Fatima Touam, Service des Maladies Infectieuses, AP-HP, Hôpital Necker, F-75015, Paris, France;

Rodolphe Thiébaut, INSERM U897, F-33076, Bordeaux, France;

Laurence Meyer, Faroudy Boufassa, Marie-Aline Charles, Rosemary Dray-Spira, Camille Legeai, Badra Boumaza, Abdellatif Essabbani, and Remonie Seng, Centre de Recherche en Epidémiologie et Santé des Populations (CESP), INSERM U1018, F-94807, Villejuif and F-94270, Le KremLin-Bicêtre, France;

Gilles Pialoux, Laurence Slama, Philippe Bonnard, Catherine Chakvetadze and Thomas L’Yavanc, Service des Maladies Infectieuses; Jacqueline Capeau, Corinne Vigouroux, Soraya Fellahi and Jean-Philippe Bastard, Service de Biochimie et Hormonologie, AP-HP, Hôpital Tenon, F-75020, Paris, France;

Jean-Paul Viard, Aline Maignan and Alain Sobel, Centre de diagnostic et de thérapeutique, AP-HP, Hôpital Hôtel Dieu, F-75001, Paris, France;

Eric Oksenhendler and Laurence Gérard, Service d’Immunopathologie Clinique; Daniel Séréni, Caroline Lascoux-Combe, Claire Pintado, Olivier Taulera, Le Van Dien, Jeannine Delgado and Sylvie Parlier, Service de Médecine Interne, AP-HP, Hôpital Saint-Louis, F-75010, Paris, France;

Jean-Michel Molina, Thierry Saint-Marc, Samuel Ferret and Juliette Pavie, Service des Maladies Infectieuses, AP-HP, Hôpital Saint-Louis and Université Diderot Paris 7, F-75010, Paris, France;

Jean-François Bergmann, Agathe Rami and Maguy Parrinello, Service de Médecine A, AP-HP, Hôpital Lariboisière, F-75010, Paris, France;

Pierre-Marie Girard, Bénédicte Lefebvre, Alysa Krain and Catherine Lupin, Service des Maladies Infectieuses, AP-HP, Hôpital Saint-Antoine, F-75012, Paris, France;

Serge Herson, Hassiba Remidi, Malik Iguertsira, , Smaïl Nafissa, Anne Simon and Nadia Edeb, Service de Médecine Interne, AP-HP, Groupe Hospitalier Pitié-Salpêtrière, F-75013, Paris, France;

Dominique Salmon-Céron, Loïc Guillevin, Tassadit Tahi and Marie Pierre Pietri, Service de Médecine Interne 2, AP-HP, Hôpital Cochin, F-75014, Paris, France;

Laurence Weiss, Marie-Laure Lucas, Magalie Ptak, Delphine Tisne-Dessus and Christelle Jalbert, Service d’Immunologie Clinique, AP-HP, Hôpital Européen Georges Pompidou, F-75015, Paris, France;

Patrick Yeni, Golriz Pahlavan, Bao Phung, Nadia El-Alami Talbi, Zahia Ramani, Giovanna Catalano, Julia Zelie, Bilguissa Diallo and Cindy Godard, Service des Maladies Infectieuses, AP-HP, Hôpital Bichat, F-75018, Paris, France;

François Boué, Véronique Chambrin, Imad kansau, Marie-Thérèse Rannou, Mariem Raho, Dominique Bornarel, Hélène Schoen, HDJ Médecine, AP-HP, Hôpital Antoine-Béclère, F-92141, Clamart, France;

Robert Carlier, Service de Radiologie et Imagerie Médicale, AP-HP, Hôpital Raymond-Poincaré, F-92380, Garches, France;

Bruno Fantin, Agnès Uludag, Ali Tadlaoui and Caroline Poder, Service de Médecine Interne, AP-HP, Hôpital Beaujon, F-92110, Paris, France;

Robin Dhote, Michelle Bentata, Tuna Lukiana and Patricia Honoré, Unité Sida; Olivier Bouchaud and Xuan Tuyet, Service des Maladies Infectieuses et Tropicales, AP-HP, Hôpital Avicenne, F-93009, Bobigny, France;

Jean-François Delfraissy, Cécile Goujard, Fabrice Chaix, Erwan Fourn and Katia Bourdic Service de Médecine Interne, AP-HP, Hôpital de Bicêtre, F-94275, Le KremLin-Bicêtre, France;

Yves Levy and Cécile Dumont, Service d’Immunologie Clinique, AP-HP, Hôpital Henri Mondor, F-94010, Créteil, France;

André Cabié, Christelle Baringthon and Véronique Beaujolais, Hôpital Pierre Zobda-Quitman, F-97261, Fort-de-France, Martinique, France;

Isabelle Poizot-Martin, Geneviève Fabre, Olivia Zaegel-Faucher, Alena Ivanova and Caroline Debreux, CISIH, Hôpital Sainte Marguerite ; Jacques Moreau, Martine Schlossers, Saadia Mokhtari and Evelyne Van Der Gheynst, Service des Maladies Infectieuses, Hôpital Nord, F-13000, Marseille, France;

Marie-Christine Thiebaut-Drobacheff, Adeline Foltzer and Catherine Bourdeaux, Service de Dermatologie, Hôpital Saint-Jacques; Bruno Hoen, Jean-François Faucher, Service des Maladies Infectieuses, Hôpital Saint-Jacques; Helder Gil, Service de Médecine Interne, Hôpital Minjoz, F-25000, Besançon, France;

Didier Neau, Charles Cazanave, Thierry Pistone, Michel Dupon, Jean-Marie Ragnaud, Isabelle Raymond, Laetitia Lacaze-Buzy and Caroline Palacin, Services des Maladies infectieuses A et B, Hôpital Pellegrin; Philippe Morlat, Sabrina Caldato, Isabelle Louis and Mojgan Hessamfar, Service de Médecine Interne et Maladies Infectieuses, Hôpital Saint-André, F-33000, Bordeaux, France;

Jacques Reynes, Claude Crisol, Vincent Baillat, Corinne Merle De Boever and Christine Tramoni, CISIH, CHRU, F-34295, Montpellier, France;

Louis Bernard, Pascale Nau, Gaëlle Sajole, Carine Sèvre, Antoine Soufflet, Patrick Guadagnin, Frédéric Bastides and Patrick Choutet, Service des Maladies Infectieuses, Hôpital Bretonneau, F-37000, Tours, France;

François Raffi, Olivier Mounoury, Véronique Reliquet, Delphine Brosseau and Hervé Hue, Service de Maladies Infectieuses, Hôpital Hôtel Dieu, F-44000, Nantes, France;

Thierry May, Amandine Briault and Simone Wassoumbou, Service des Maladies infectieuses, Hôpital Brabois, CHU de Nancy, F-54511, Vandoeuvre-les-Nancy, France;

Antoine Chéret, Séverine Bonne, Yazdan Yazdanpanah, Thomas Huleux and Emmanuelle Aïssi, Service des Maladies Infectieuses, Hôpital Gustave Dron, F-59000, Tourcoing, France;

David Rey and Christine Cheneau, Le Trait d'Union Centre de Soins de l'Infection par le VIH, NHC, Hôpital Civil, F-67000, Strasbourg, France;

Mahsa Mohseni Zadeh, Aurélie Richard, Gilles Blaison, Martin Martinot and Anne Pachard, Service d’Onco-Hématologie, Hôpital Pasteur, F-68000, Colmar, France;

Djamila Makhloufi, François Jeanblanc and Jean-Louis Touraine, Service d’Immunologie, Hôpital Edouard Herriot ; Laurent Cotte, Claude Augustin-Normand, Joseph Koffi Patrick Miailhes, Sophie Pailhes and Isabelle Schlienger Service de Maladies Infectieuses et Tropicales, Hôpital de La Croix-Rousse, F-69000, Lyon, France

Philippe Perré, Jean Luc Esnault and Isabelle Suaud, Service de Médecine Interne, Centre Hospitalier Départemental, F-85000, La Roche sur Yon, France.
